# Publisher Correction: Mammalian display screening of diverse cystine-dense peptides for difficult to drug targets

**DOI:** 10.1038/s41467-018-03350-5

**Published:** 2018-03-09

**Authors:** Zachary R. Crook, Gregory P. Sevilla, Della Friend, Mi-Youn Brusniak, Ashok D. Bandaranayake, Midori Clarke, Mesfin Gewe, Andrew J. Mhyre, David Baker, Roland K. Strong, Philip Bradley, James M. Olson

**Affiliations:** 1 Clinical Research Division, Fred Hutchinson Cancer Research Center, 1100 Fairview Avenue N Room D4-100, Seattle, WA 98109 USA; 2 Basic Sciences, Fred Hutchinson Cancer Research Center, 1100 Fairview Avenue N Room B3-183, Seattle, WA 98109 USA; 3 Department of Biochemistry, University of Washington, Molecular Engineering and Sciences, Box 351655, Seattle, WA 98109 USA; 4 Public Health Sciences, Fred Hutchinson Cancer Research Center, 1100 Fairview Avenue N Room M1-B514, Seattle, WA 98109 USA

Correction to: *Nature Communications* 10.1038/s41467-017-02098-8, published online 21 December 2017

In the original version of this Article the colour key for the amino acid enrichment score was inadvertently omitted from the lower panel of Figure 5b during the production process. This has now been corrected in the PDF and HTML versions of the Article.

