# Supplementary material for: Mammalian display screening of diverse cystine-dense peptides for difficult to drug targets
Source: Nat Commun. 2017 Dec 21;8:2244. doi: 10.1038/s41467-017-02098-8 (PMC5740061; doi:10.1038/s41467-017-02098-8)
Supplement: Supplementary file 1 — Supplementary Information [file 41467_2017_2098_MOESM1_ESM.docx]

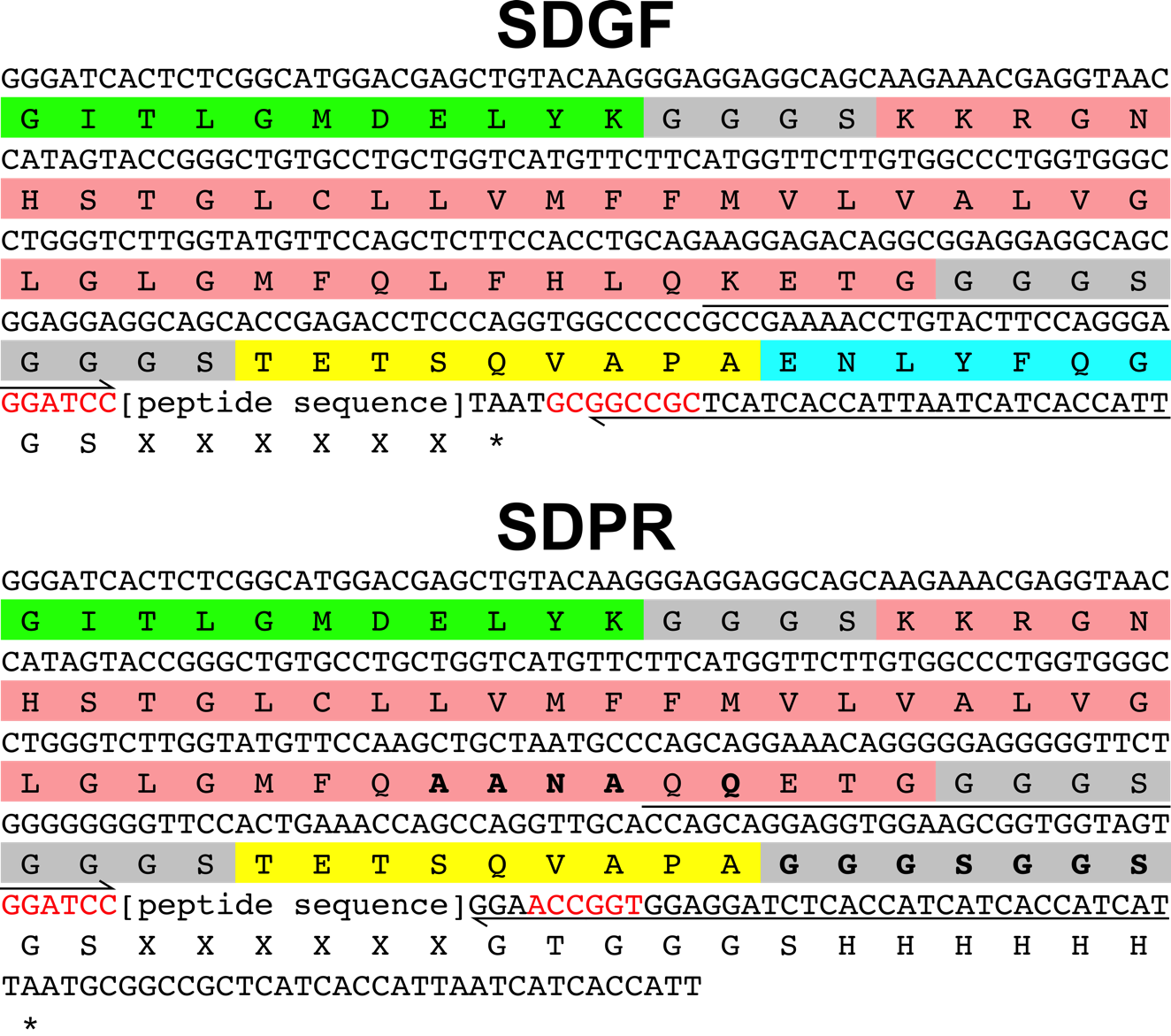
 **Supplementary Figure 1 | SDGF and SDPR vector maps.** Shown are the portions of the vectors relevant to display and cloning: the C-Terminal portion of GFP, FasL transmembrane domain, tags, cloning sequences, and flanking vector sequences. Green, GFP; Pink, FasL transmembrane domain; yellow, bovine rhodopsin C9 tag; blue, TEV protease cleavage recognition site; grey, linker sequences. Bold protein text in SDPR highlights mutations between SDGF and SDPR, eliminating trypsin and chymotrypsin sensitive sites (basic and aromatic residues, respectively). Red DNA text indicates relevant restriction enzyme cut sites for opening the vectors to facilitate peptide cloning. Half arrowhead lines indicate vector-overlapping primer sequences used for Gibson assembly.


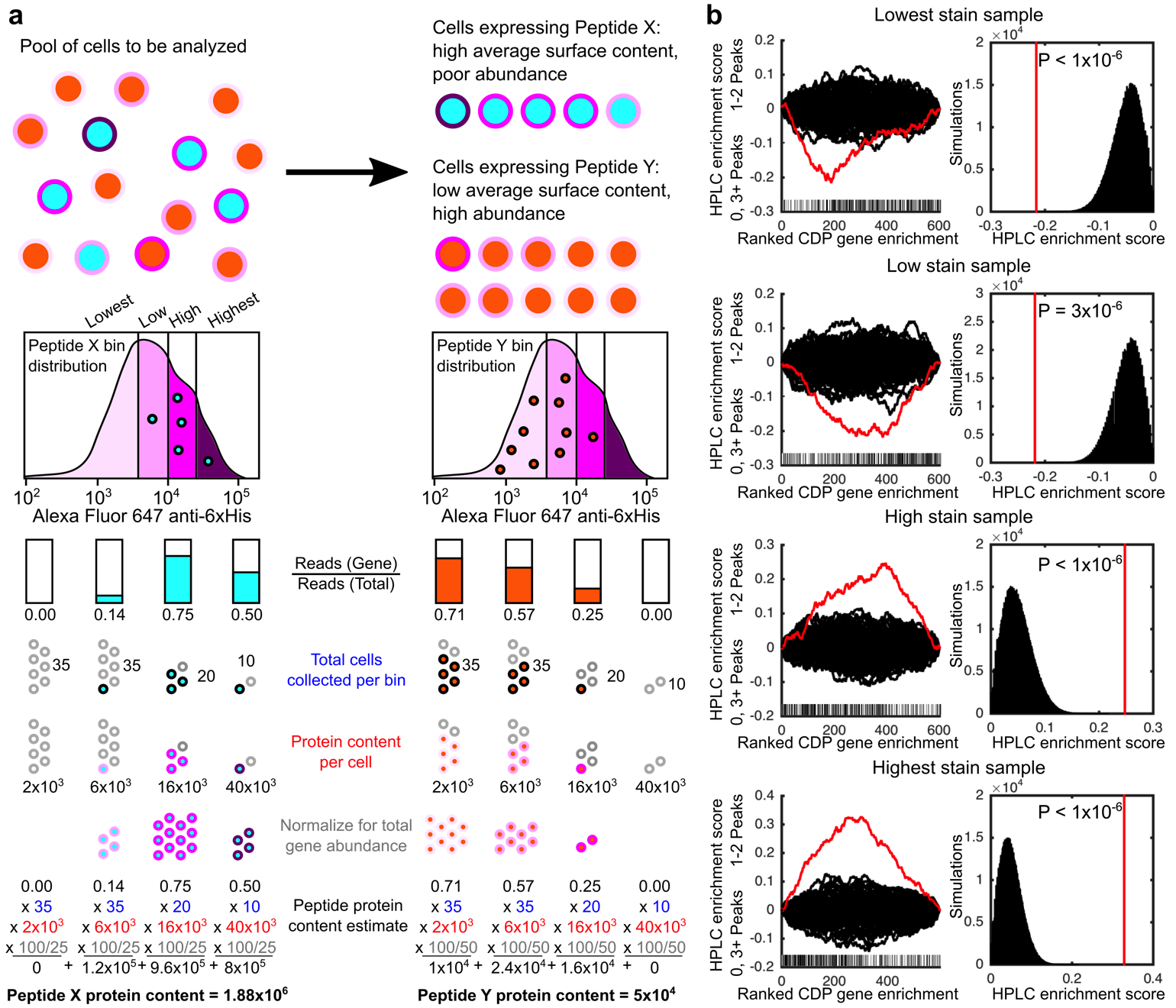


**Supplementary Figure 2 | Computational method for assigning peptide-by-peptide surface protein content in a pooled library.** (**a**) In a pool of cells expressing different surface-displayed peptides, some peptides may confer high average surface staining (Gene X); others, low staining (Gene Y), as illustrated by the purple border darkness. Gene abundance will also vary. For the two representative genes, flow sorting into four samples will distribute them mainly into either the high stain (Gene X) or low stain (Gene Y) samples. Upon sequencing, read abundance will be converted into a surface protein content estimate for each sample by accounting for: 1) the actual number of cells collected in each sample (blue); 2) the median protein content for a cell in each of the samples (red); and 3) for the total abundance of cells expressing that gene in the treatment condition (grey). These per-sample content estimates are summed to generate a total protein content estimate for each peptide, and this is performed in untreated and trypsin-treated conditions. (**b**) Demonstration of well-folded (by HPLC) CDP enrichment only among higher staining cells in the untreated population. For each of the four sorted samples, CDP gene enrichment was calculated vs. the input population, then ranked (descending). An unweighted gene set enrichment analysis (GSEA) on the HPLC-validated peptides, measuring enrichment of 1-2 Peak peptides, was performed on the ranked list for each sample. Left panels: the actual GSEA plot (red) along with 100 plots generated by shuffled simulations (black). Black vertical stripes above the X axis indicate 1-2 Peak peptides at that location of the ranked list; white stripes are 0 or 3+ Peak peptides. Right panels: histogram of peak enrichment scores generated by 1,000,000 shuffled simulations. The red line is at the real peak enrichment score. P-value represents the fraction of shuffled simulations that produced a more extreme peak than the real enrichment score.


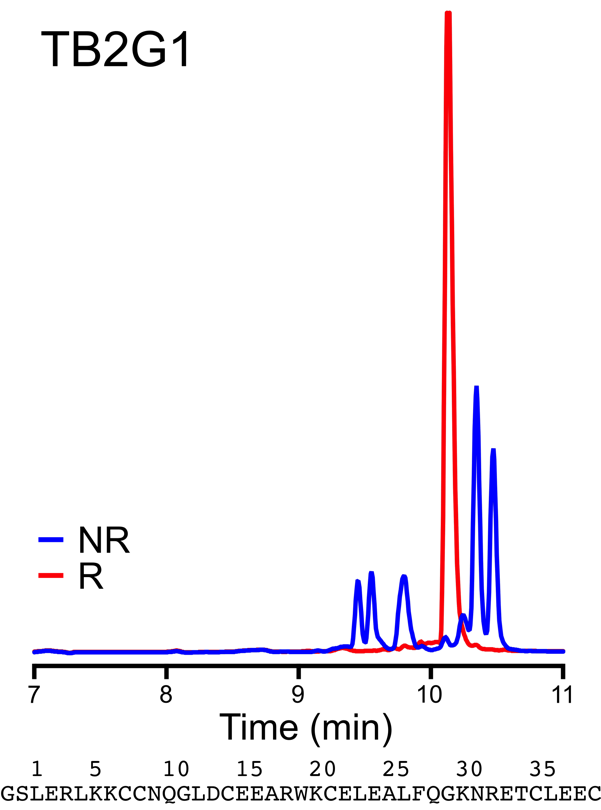


**Supplementary Figure 3 | TB2G1 soluble peptide has multiple disulfide connection patterns.** The reversed-phase HPLC traces of the non-reduced (NR) and 10 mM DTT-reduced (R) TB2G1 peptide indicate multiple species prior to reduction. Because they have the same mobility upon elimination of disulfide bonds, we can conclude that there is only one peptide product, and that the different species are the peptide existing in different disulfide connection patterns.


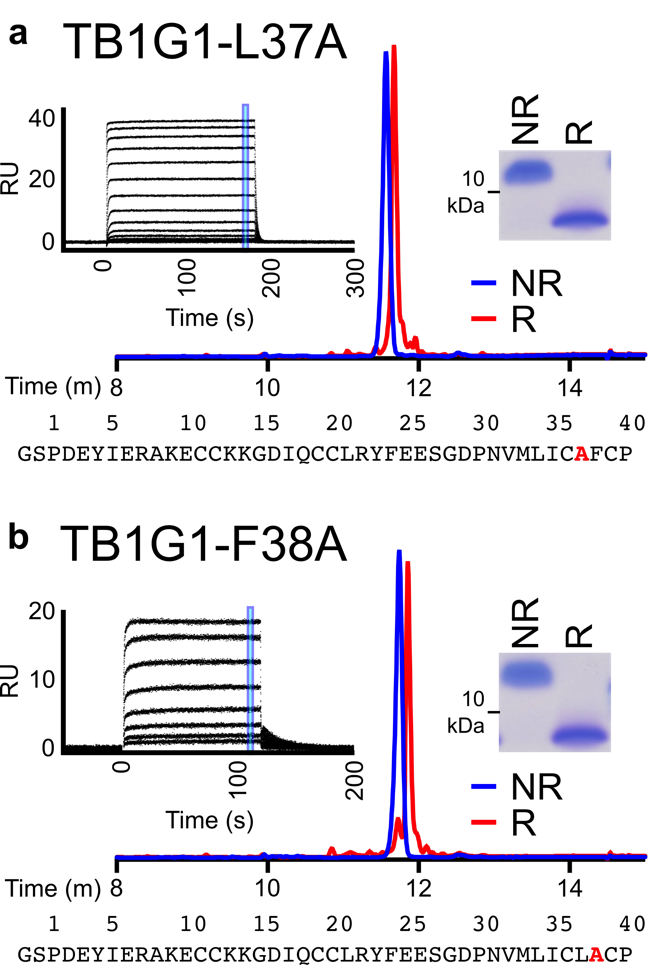


**Supplementary Figure 4 | Effect of predicted binding site mutations on TB1G1 TEAD-binding.** Like TB1G1, the L37A (**a**) and F38A (**b**) variants of TB1G1 are also monodisperse proteins that exhibit mobility shifts upon reduction in both HPLC (bottom of each panel) and SDS-PAGE (right insets). Mutated residues from TB1G1 are in bold, red text. Equilibrium binding constants (*K*_D_) determined by SPR for TB1G1-L37A and TB1G1-F38A binding to TEAD were 0.28 ± 0.02 µM and 22.8 ± 0.5 µM, respectively (L37A: serial 2-fold dilutions of 10 μM 🡪 0.6 nM in duplicate; F38A: serial 2-fold dilutions of 125 μM 🡪 980 nM in duplicate). For both panels, equilibrium SPR responses were measured in the region in blue; steady-state curves fitted to equilibrium SPR responses are shown in **Supplementary Fig. 8b,c**. Please see **Methods** and **Supplementary Table 4** for SPR methodology and analytical models.


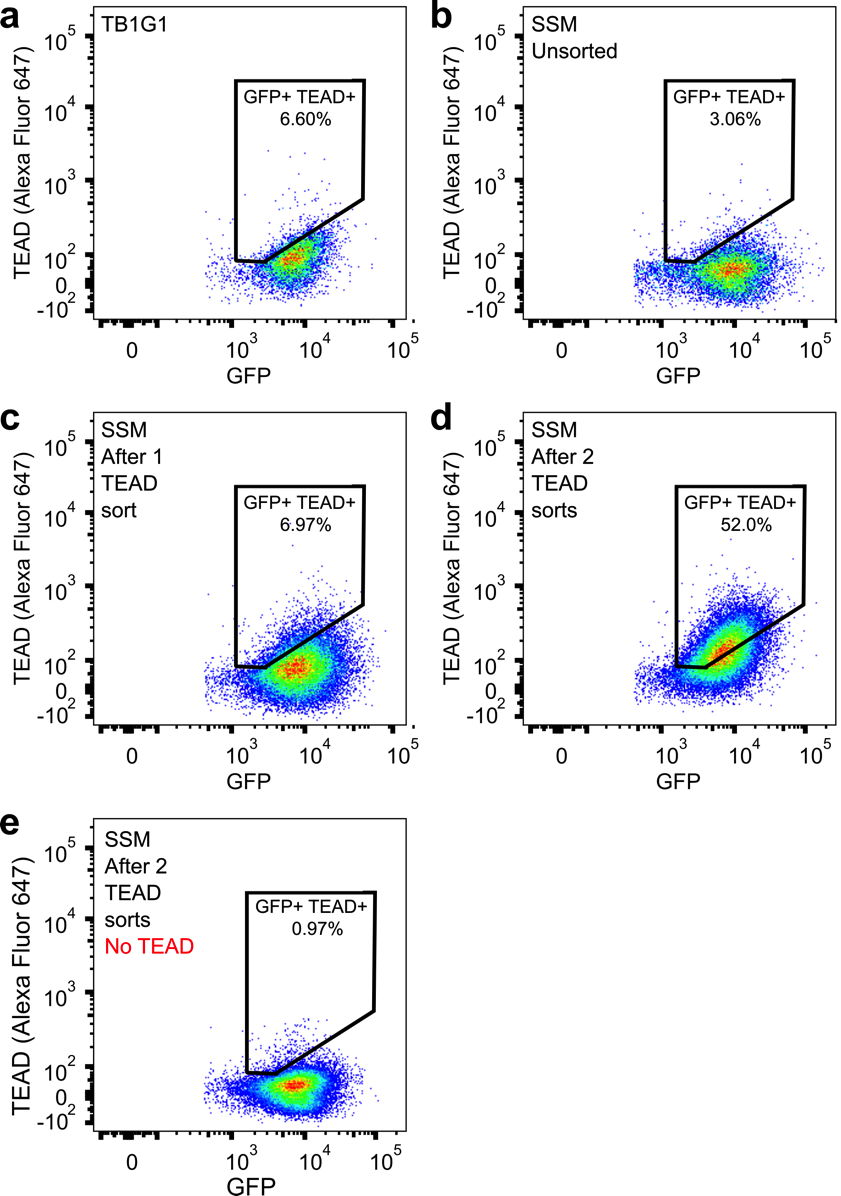


**Supplementary Figure 5 | A site saturation mutagenesis (SSM) library of TB1G1 variants contains improved TEAD binders.** (**a**) TB1G1 binding is apparent but weak under more stringent binding conditions (20 nM TEAD concentration, 2-step staining). (**b-d**) The SSM library, either unsorted (**b**), sorted once (**c**), or sorted twice (**d**), and stained with TEAD followed by streptavidin-Alexa Fluor 647. (**e**) Binding profile of the population in (**d**) stained with streptavidin-Alexa Fluor 647 only, without TEAD.


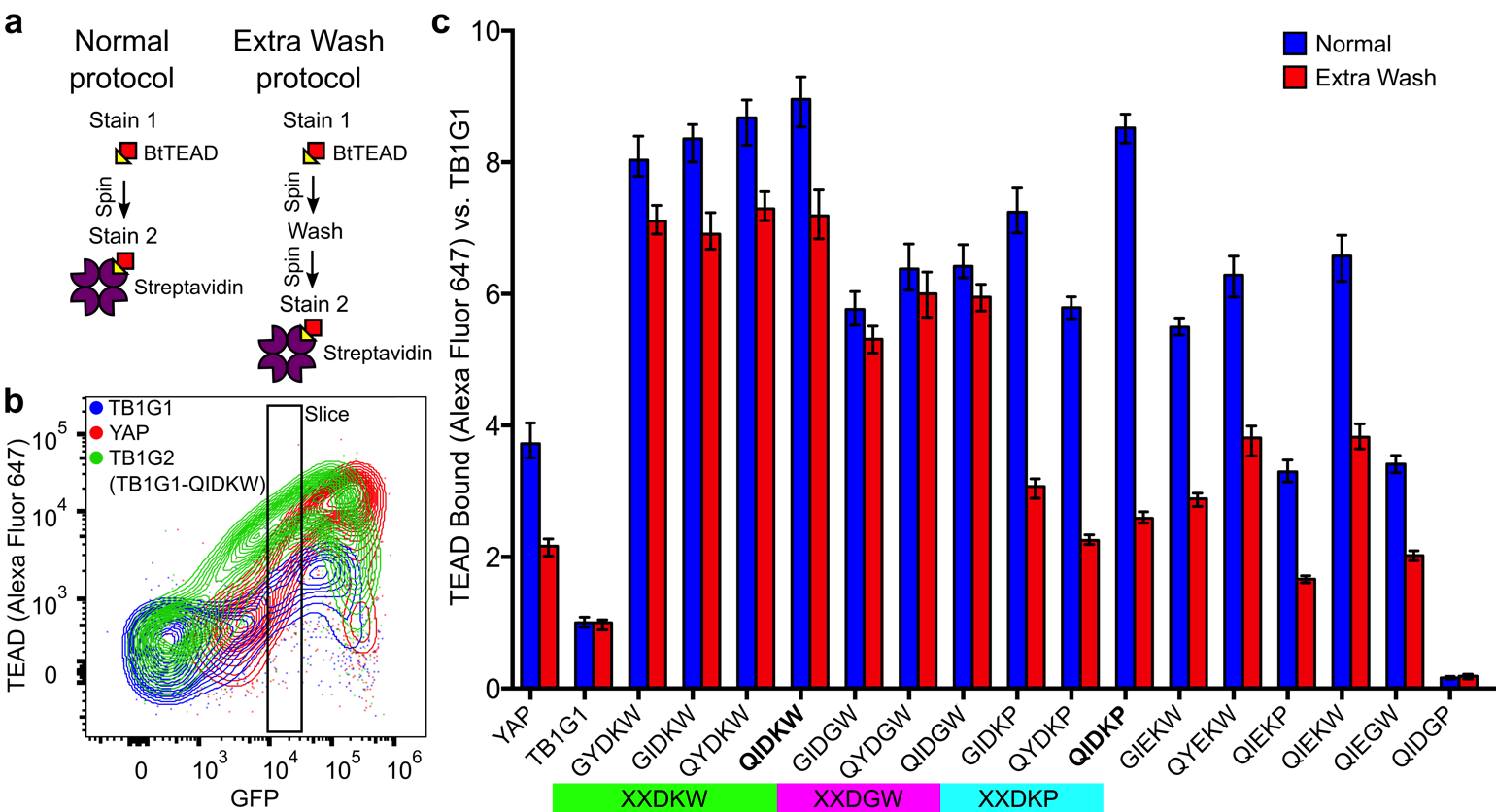


**Supplementary Figure 6 | Combining beneficial substitutions to improve upon TB1G1 TEAD binding.** (**a**) Binding of improved TB1G1 variants to TEAD was tested under more stringent conditions than were used in the primary screen. Cells displaying TEAD binders were incubated with a lower concentration (20 nM) of biotinylated TEAD (BtTEAD), then pelleted and either resuspended in buffer with streptavidin-Alexa Fluor 647 (Normal protocol), or washed with PBS once before streptavidin staining (Extra Wash protocol). (**b**) Flow cytometry binding profile of 293F cells expressing TEAD binders cloned into SDGF, under the Extra Wash protocol from (**a**). TB1G2 is a quintuple mutant of TB1G1. (**c**) Five substitutions were tested for their effect on TEAD binding as triple, quadruple, or quintuple mutant variants of TB1G1: G15Q, Y23I, E25D, G28K, and P40W. Five-letter codes below the chart represent the residues used at these five sites. All variants were cloned into SDGF and tested for binding to biotinylated TEAD with both the Normal protocol and the Extra Wash protocol. Binding was quantitated by the Alexa Fluor 647-streptavidin signal in cells within the narrow “slice” gate, seen in (**b**). Shown are the median +/- 95% confidence intervals, normalized to that of TB1G1. Note that one substitution, P40W, was particularly crucial for improving binding when an extra wash was performed (XXDKW [green] vs XXDKP [cyan]), which may indicate an improvement of the off-rate. Variants in bold (QIDKW and QIDKP) were selected for soluble protein production as TB1G2 and TB1G2-W40P, respectively.


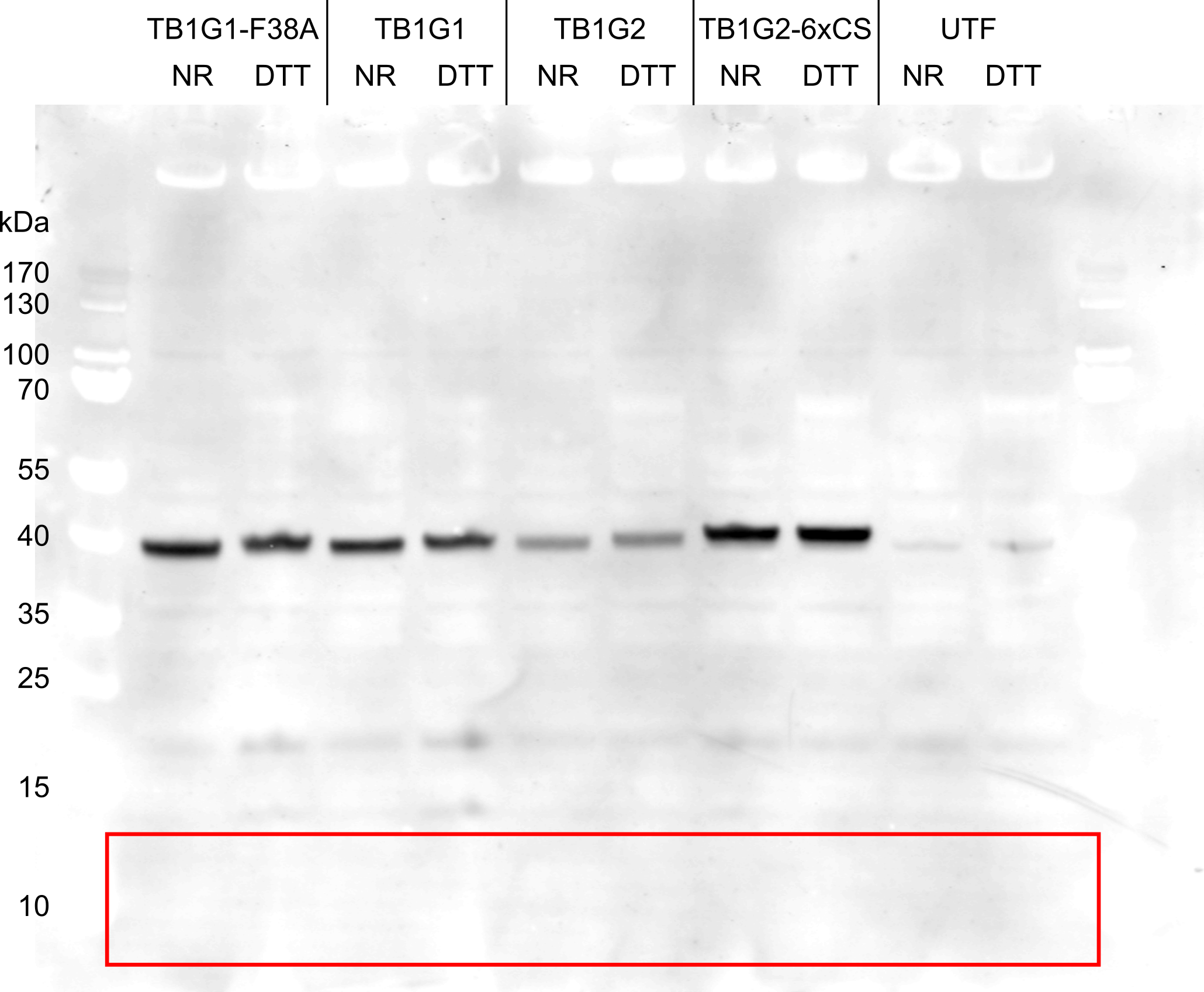


**Supplementary Figure 7 | An mCherry-T2a-FLAG-optide construct shows intact fusion but not cleaved, soluble optides in 293T cell lysate.** Shown is the full anti-FLAG M2 Western blot from **Fig. 7i**, with mild contrast enhancement. The prominent bands correspond to the expected size of the intact mCherry-T2a-optide. The red box shows the region where T2a-cleaved, FLAG-tagged optides would be expected to run, based on SDS-PAGE of soluble, untagged optides. NR: non-reduced. DTT: 5 mM (final) DTT, added immediately before loading the SDS-PAGE gel. 6xCS: all six cysteines were mutated to serines, to simulate cysteines that cannot be oxidized to cystines. UTF: Untransfected 293T lysate.


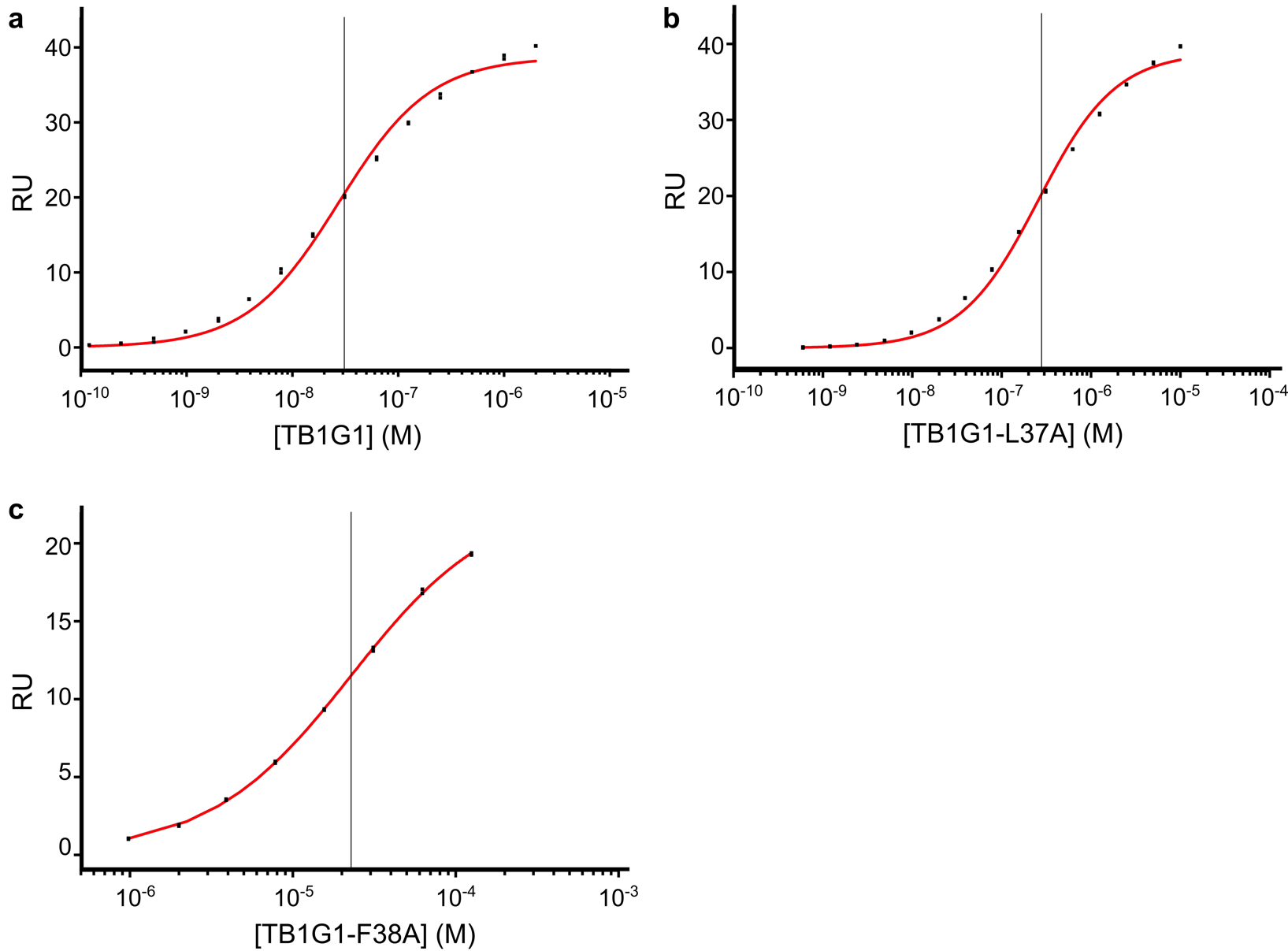


**Supplementary Figure 8 | Fitting of SPR equilibrium responses with 1:1 binding models.** Used for determining *K*_D_ values for the binding of TB1G1 and its L37A and F38A variants to TEAD. Equilibrium measurements were used to characterize these interactions because the interaction kinetics were too fast to be accurately determined. (**a**) TB1G1. (**b**) TB1G1-L37A. (**c**) TB1G1-F38A. Grey line = *K*_D_. RU = SPR response units.


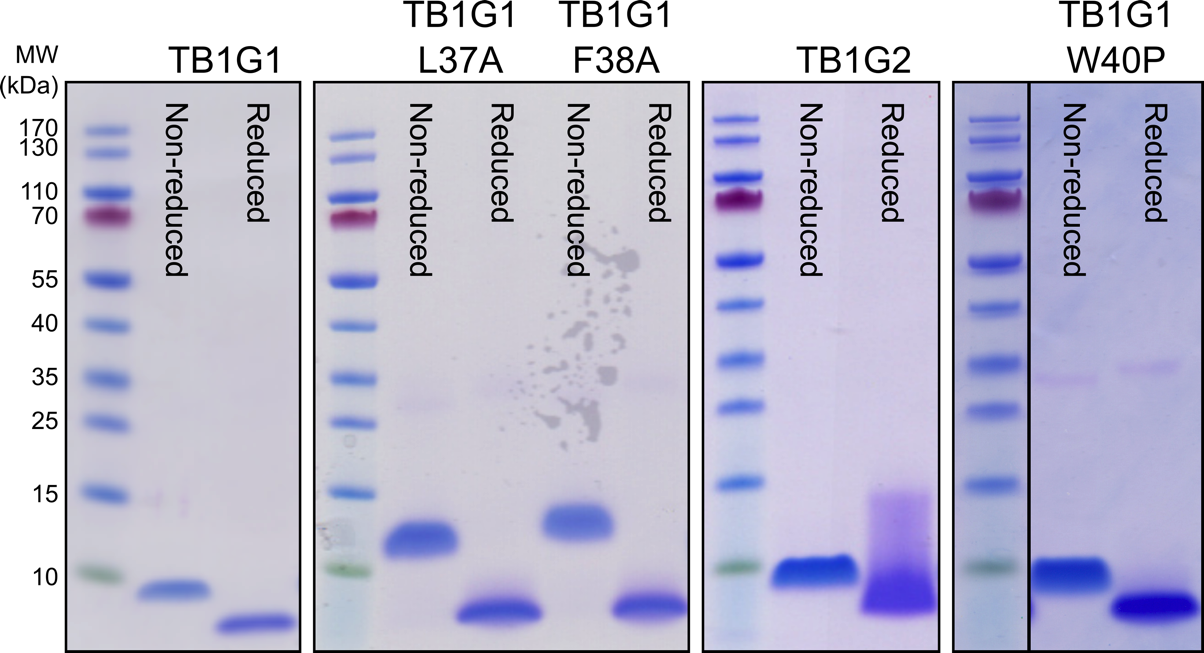


**Supplementary Figure 9 | Full Coomassie gels of TEAD-binding Optides.** Gels were cropped to produce insets to **Fig. 4h**, **Supplementary Fig. 4a,b**, and **Fig. 5c,d**. Reduction was accomplished by incubation with 5 mM DTT. Note that, while the mobility of the non-reduced optides relative to the SDS-PAGE ladders may suggest molecular weights above that predicted by amino acid sequence (particularly for non-reduced samples), the reduced (i.e. linearized) samples ran where expected. Furthermore, mass spectrometry confirmed proper molecular weight of TB1G2 (see **Fig. 6a** and **Supplementary Fig. 10**). In general, this behavior is commonplace for CDPs in our hands. It may suggest inaccurate mobility of the pre-stained markers, or could be the result of reduced SDS saturation relative to molecular weight for intact, non-reduced CDPs.


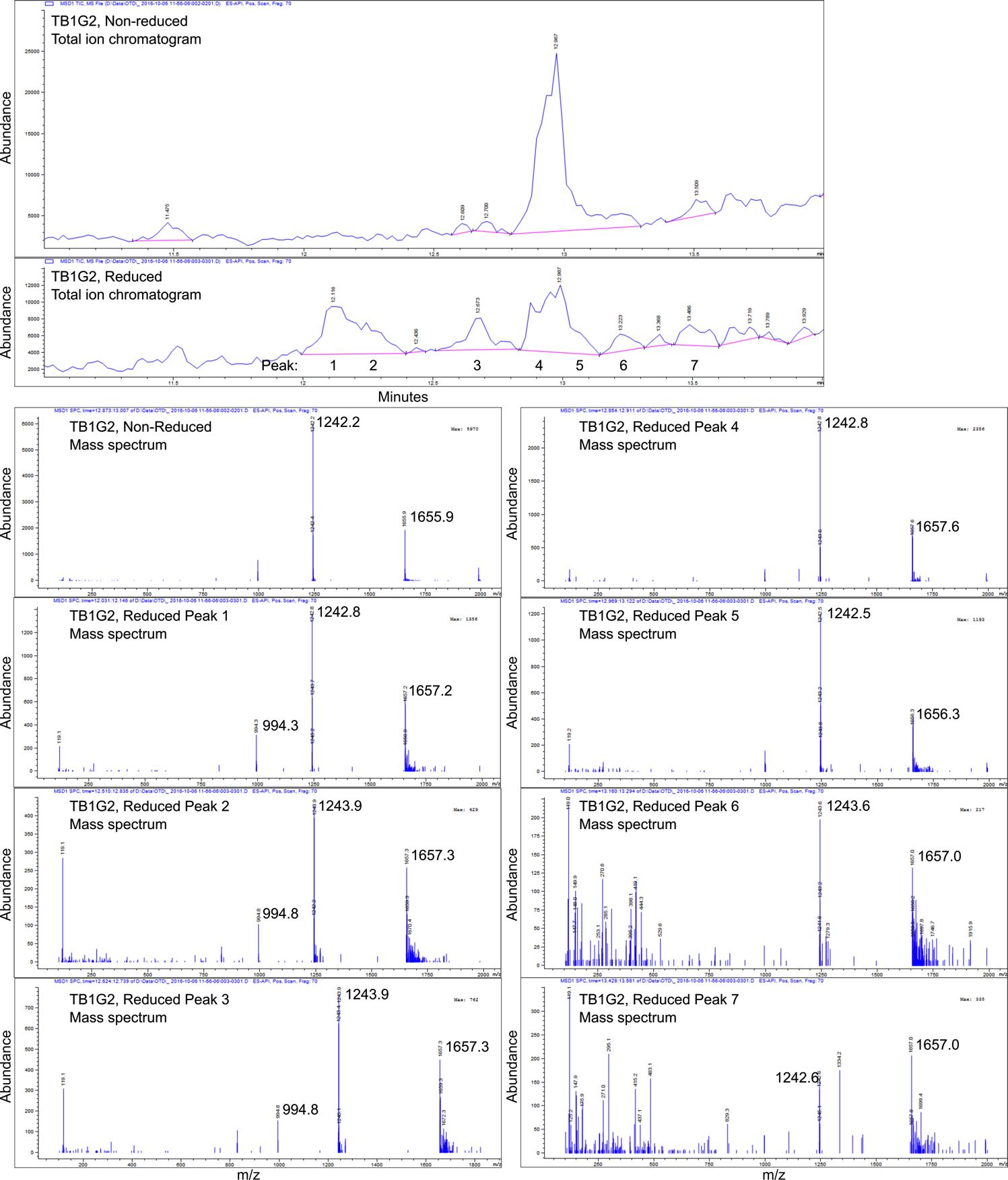


**Supplementary Figure 10 | Mass spectra of non-reduced and reduced TB1G2.** Spectra represent the complete MS dataset shown in part in **Fig. 6a**. The m/z peaks of ~995, ~1244, and ~1657 represent the +5, +4, and +3 charged peptide, respectively.


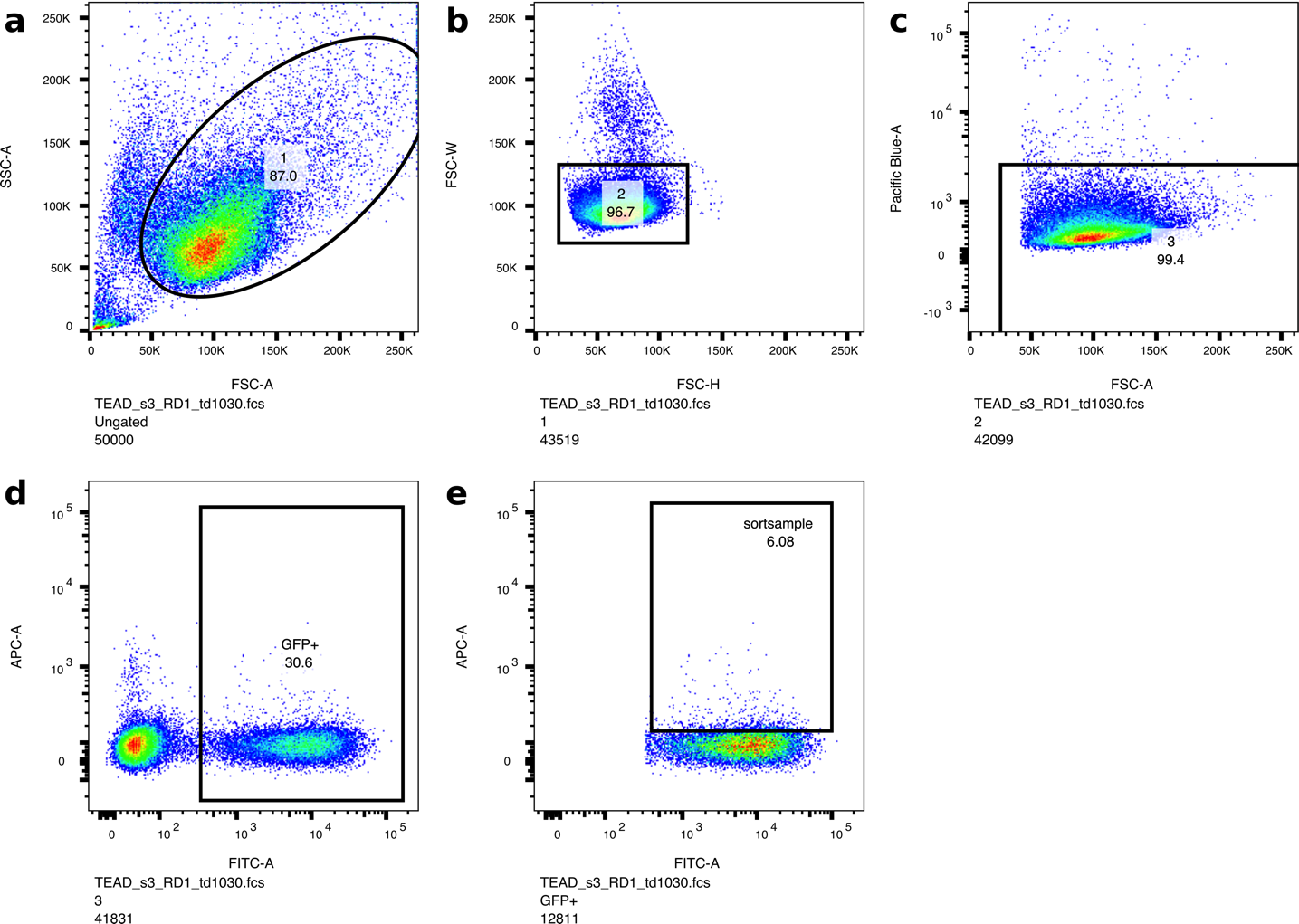


**Supplementary Figure 11 | Example of gating process for cells sorted on BD Aria II instrumentation.** Sample represents the input material to the first TEAD binder sort, seen in **Figure 3b**, top left panel. (**a**) All events, FSC-A vs SSC-A, elliptical Gate 1. (**b**) Gate 1, FSC-H vs FSC-W, rectangular Gate 2. (**c**) Gate 3, FSC-A vs Pacific Blue-A (DAPI), rectangular Gate 3. (**d**) Gate 3, FITC-A (GFP) vs. APC-A (TEAD AlexaFluor 647), rectangular GFP+ gate. (**e**) GFP+ gate, FITC-A (GFP) vs. APC-A (TEAD AlexaFluor 647), rectangular APC+ gate (maintained at ~7% of GFP+ events for all screens; this value can be adjusted to the user’s stringency requirements).


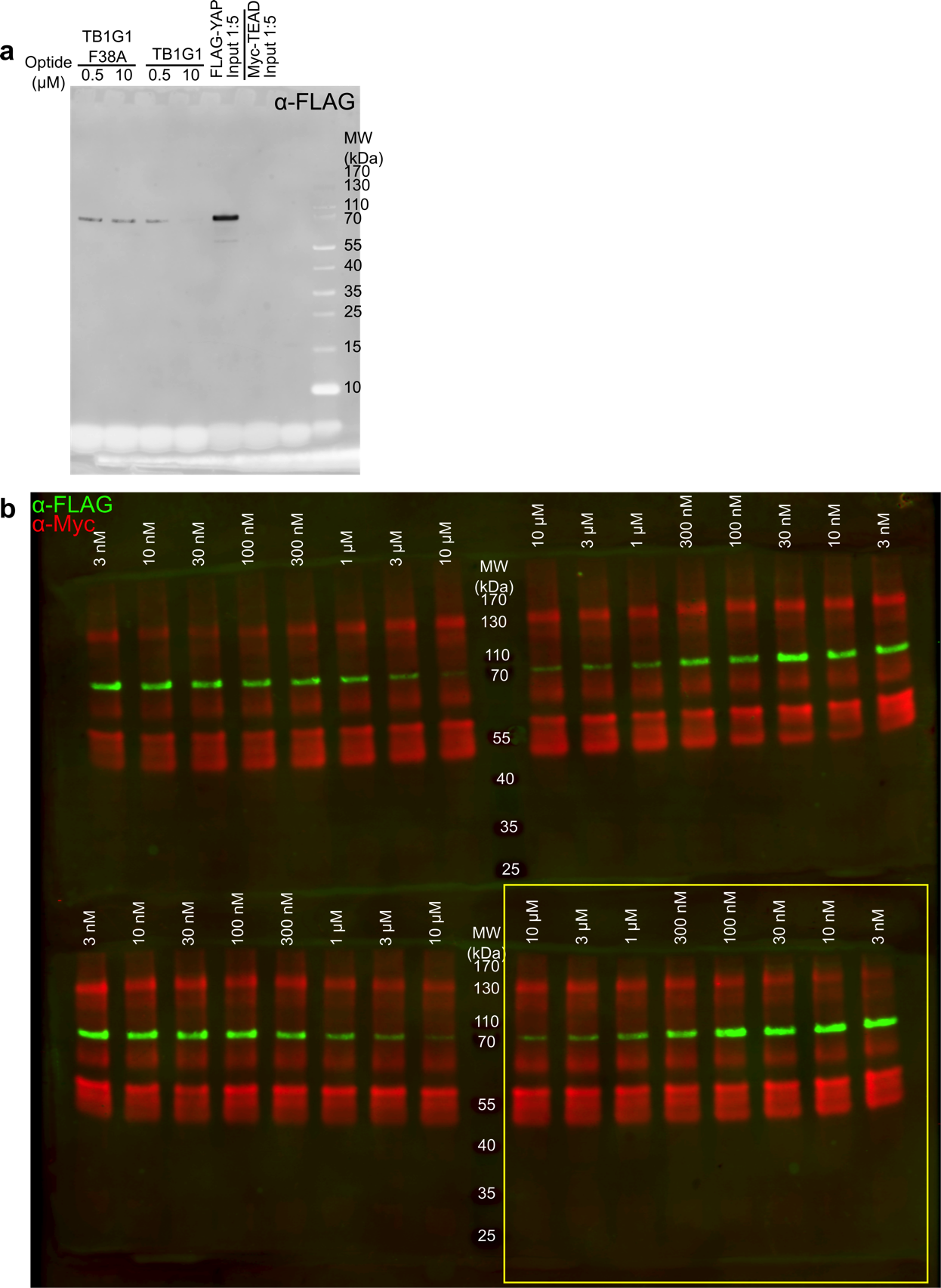


**Supplementary Figure 12 | Full Western blots of YAP:TEAD co-immunoprecipitation.** Figures represent blots from **Fig. 4i** (**a**) and **Fig. 4j,k** (**b**). Note: all four dilution series were quantitated (after splitting channels) to create the data in **Fig. 4k**, but only the bottom right dilution series (yellow border) from the blot was used for **Fig. 4j** (flipped horizontally and cropped to the relevant masses).

| **Species** | **Secretome Size (Predicted)** | **# (%) with**  **C-rich Domain** | **Database** |
| --- | --- | --- | --- |
| *E. coli* (K12) | 1526 proteins | 11 (0.7%) | http://www.stepdb.eu/step2/exportomelist.php?cmd=reset |
| *S. cerevisiae* | 1268 proteins | 42 (3.3%) | http://bioinformatics.ysu.edu/secretomes/fungi2/index.php |
| *H. sapiens* | 7439 genes | 1452 (19.5%) | http://www.proteinatlas.org/humanproteome/secretome |

**Supplementary Table 1 | Secreted proteins with cysteine-rich domains for *E. coli*, *S. cerevisiae*, and *H. sapiens*.** The respective secretomes, including membrane proteins, were downloaded from the indicated databases. For *E. coli*, the exportome was searched, and for *S. cerevisiae*, “likely secreted”, “highly likely secreted”, and “curated secreted” proteins were searched. For *H. sapiens*, all predicted membrane and/or secreted genes were searched. Protein sequences with the ambiguous amino acid “X” were omitted. Ensembl Gene IDs (human) or UniProt accession numbers (*E. coli* and *S. cerevisiae*) were converted to consensus protein sequences from UniProt (http://www.uniprot.org/uploadlists/ , converted to UniProtKB), which were searched by a recursive script to identify protein sequences that contain at least 6 C in a span of 44 amino acids. For *H. sapiens*, all protein isoforms were searched, but then all protein IDs were converted to unique gene IDs before counting, so as to not inflate counts due to splice isoforms. Gene ID conversions were performed on the DAVID Bioinformatics Resources online tool (https://david.ncifcrf.gov/conversion.jsp).

| **Plot** | **HPLC Peaks** | **Knottin or Defensin** | **Protein Content** | **Trypsin Resistance** | **UniProt** | **Protein** | **Species** | **Sequence** |
| --- | --- | --- | --- | --- | --- | --- | --- | --- |
| 1 | 1-2 | Yes | 44466 | 90 | Q2V3S8 | Putative defensin-like protein 257 | *Arabidopsis thaliana* | GSAPPCKRDVDCSFECPKGGFCNDRLGTCDCF |
| 2 | 1-2 | Yes | 40248 | 84 | P0C8B2 | Venom defensin-like peptide A | *Ornithorhynchus anatinus* | GSRQPCSYYDGVCRDKSDVNCKYIAFTYCENPNQRCCYY |
| 3 | 1-2 | Yes | 39568 | 102 | Q5Y4U6 | Beta/delta-agatoxin-6 | *Agelena orientalis* | GSERECVGENGHCRSWYNDCCDGYYCSCMQPPNCICRNN |
| 4 | 3+ | Yes | 40158 | 95 | Q8NET1 | Beta-defensin 108B | *Homo sapiens* | GSKEICERP**NGS**CRDFCLETEIHVGRCLNSQPCCLPL |
| 5 | 1-2 | No | 44977 | 75 | P09944 | Hirudin-PA | *Hirudo medicinalis* | GSYTDCTESGQNLCLCEGSNVCGKGNKCILGSQGKDNQCVTG |
| 6 | 1-2 | No | 40219 | 97 | P26886 | Mating pheromone Er-2/Er-9 | *Euplotes raikovi* | GSPMTCEQAMASCEHTMCGYCQGPLYMTCIGITTDPECGLP |
| 7 | 1-2 | No | 38232 | 96 | R0FZM9 | Uncharacterized protein | *Capsella rubella* | GSKDYCPGICNVAAVPDCDTLCISLGYSGGYCRAGRICCCNPK |
| 8 | 3+ | No | 37742 | 96 | D5KXG8 | Turripeptide IX-01 | *Gemmula speciosa* | GSYASCEAAEADCIHDDCFSEDTYTDVCQESCQYMYDNCMDD |
| 9 | 1-2 | Yes | 9071 | 65 | P80223 | Corticostatin-6 | *Oryctolagus cuniculus* | GSGICACRRRFCLNFEQFSGYCRVNGARYVRCCSRR |
| 10 | None | Yes | 12770 | 43 | P50711 | Alpha-defensin 13 | *Mus musculus* | GSDLVCYCRKRGCKRREHM**NGT**CRRGHLMYTLCCR |
| 11 | None | Yes | 9788 | 46 | B1P1C7 | U9-theraphotoxin-Cj1a | *Chilobrachys guangxiensis* | GSERGCGLLMDACDGKSTFCCSGY**NCS**PTWKWCVLDCPNLFLLPPTKTLC |
| 12 | 3+ | Yes | 10083 | 60 | A1YL78 | Agouti-signaling protein | *Callithrix geoffroyi* | GSSVPCVSTRGSCKPPAPACCHPCASCQCRFFRSACSCRVLNVNC |
| 13 | 1-2 | No | 16688 | 52 | E3NBU0 | Putative uncharacterized protein | *Caenorhabditis remanei* | GSRGQCWSYSNCRAVCRDEGYVSGHCNYFGGACWCAS |
| 14 | 3+ | No | 10056 | 34 | O42236 | Semaphorin-3C | *Gallus gallus* | GSLHRCRIYGTACADCCLARDPYCAWDGNSCSRF |
| 15 | 3+ | No | 8771 | 26 | I0CME7 | Hyaluronidase conohyal-Cn1 | *Conus consors* | GSSDLCSTRQSRFRDYHCRCYSAWEGACCQTLRPSRCQKR |
| 16 | 3+ | No | 8032 | 50 | Q2KJ63 | Plasma kallikrein | *Bos taurus* | GSVDGCQETCTKMIRCQFFTYSLFPEDCRGEKCKCSLR |

**Supplementary Table 2 | Representative peptides within surface displayed CDP diversity library.** These are presented as a companion to the soluble HPLC plots in **Fig. 2c**. Predicted N-linked glycosites (NXS/T) are in bold, underlined font.

| Peptide:Full Protein | < 25% | | | | 25-50% | | | | > 50% | | | |
| --- | --- | --- | --- | --- | --- | --- | --- | --- | --- | --- | --- | --- |
| HPLC Peaks | No Data | 0 Peaks | 3+ Peaks | 1-2 Peaks | No Data | 0 Peaks | 3+ Peaks | 1-2 Peaks | No Data | 0 Peaks | 3+ Peaks | 1-2 Peaks |
| LC/TS,  0 Glycosites | 2092 | 4 | 23 | 4 | 324 | 18 | 81 | 44 | 111 | 26 | 76 | 48 |
| LC/TS,  1+ Glycosites | 372 | 0 | 1 | 0 | 51 | 9 | 8 | 5 | 21 | 2 | 2 | 4 |
| HC/TR,  0 Glycosites | 223 | 1 | 1 | 7 | 65 | 4 | 19 | 61 | 46 | 2 | 36 | 85 |
| HC/TR,  1+ Glycosites | 98 | 0 | 0 | 0 | 11 | 0 | 11 | 5 | 10 | 0 | 8 | 6 |

**Supplementary Table 3 | Distribution of displayed peptides by surface folding, secreted structural homogeneity (HPLC peaks), peptide:full protein ratio, and glycosite occurrence.** HC/TR: High surface content and/or trypsin resistant. LC/TS: Low surface content and/or trypsin sensitive. Inclusive of peptides that passed read abundance thresholds and had reliable UniProt data for consensus full protein context. Full table of peptide characteristics is available as **Supplementary Data 3**.

| **Analyte** | ***k*_a_ (M^-1^s^-1^)** | | ***k*_d_ (s^-1^)** | | ***K*_D_ (nM)** | | | **Sequence** | | | |  |  |
| --- | --- | --- | --- | --- | --- | --- | --- | --- | --- | --- | --- | --- | --- |
|  |  |  |  |  |  |  |  |  |  |  |  |  | |
| **TB1G1** | ND | | ND | | 31 ± 2 | | | GSPDEYIERAKECCKKGDIQCCLRYFEESGDPNVMLICLFCP | | | |  |  |
|  |  |  |  |  |  |  |  |  |  |  |  |  | |
| **TB1G1-L37A** | ND | | ND | | 280 ± 20 | | | GSPDEYIERAKECCKKGDIQCCLRYFEESGDPNVMLICAFCP | | | |  |  |
|  |  |  |  |  |  |  |  |  |  |  |  |  | |
| **TB1G1-F38A** | ND | | ND | | 22800 ± 500 | | | GSPDEYIERAKECCKKGDIQCCLRYFEESGDPNVMLICLACP | | | |  |  |
|  |  |  |  |  |  |  |  |  |  |  |  |  | |
| **TB1G2** | 2.21 ± 0.02 x10^7^ | | 8.14 ± 0.06 x10^-3^ | | 0.368 ± 0.004 | | | GSPDEYIERAKECCKKQDIQCCLRIFDESKDPNVMLICLFCW | | | |  |  |
|  |  |  |  |  |  |  |  |  |  |  |  |  | |
| **TB1G2-W40P** | 3.10 ± 0.03 x10^7^ | | 0.117 ± 0.001 | | 3.78 ± 0.05 | | | GSPDEYIERAKECCKKQDIQCCLRIFDESKDPNVMLICLFCP | | | |  |  |
|  |  |  |  |  |  |  |  |  |  |  |  |  | |
| **Analyte** | **Ligand: Captured Biotinylated TEAD** | | | | | **Analyte: TEAD Binder** | | | | | | |  |
|  | **Reference: Captured Biotinylated hTfR(ED)** | | | | |  |  |  |  |  |  |  |  |
|  | **Flow Rate (µL min^-1^)** | **Concentration (µg mL^-1^)** | **Injection Time (s)** | **Capture Level (RU)** | | **Flow Rate (µL min^-1^)** | **Concentration** | | **Injection Time (s)** | **Dissociation Time (s)** | **Analysis Model** | |  |
|  |  |  |  |  |  |  |  |  |  |  |  |  |  |
| **TB1G1** | 10 | 1.7 | 430 | 330 | | 50 | 2 μM → 0.12 nM in duplicate, serial 2x dilutions, randomly injected | | 180 | 180 | Steady-State | |  |
|  | 10 | 2 | 390 | 882 | |  |  |  |  |  |  |  |  |
| **TB1G1-L37A** | 10 | 1.7 | 430 | 330 | | 50 | 10 μM → 0.6 nM in duplicate, serial 2x dilutions, randomly injected | | 180 | 180 | Steady-State | |  |
|  | 10 | 2 | 390 | 882 | |  |  |  |  |  |  |  |  |
| **TB1G1-F38A** | 10 | 1.7 | 430 | 330 | | 50 | 125 μM → 980 nM in duplicate, serial 2x dilutions, random injections | | 120 | 120 | Steady-State | |  |
|  | 10 | 2 | 390 | 882 | |  |  |  |  |  |  |  |  |
| **TB1G2** | 10 | 2 | 60 | 250 | | 50 | 0.044, 0.133, 0.4, | | 420 | 900 | Single Cycle Kinetics | |  |
|  | 10 | 2 | 84 | 700 | |  | 1.2, and 3.6 nM | |  |  |  |  |  |
| **TB1G2-W40P** | 10 | 2 | 60 | 250 | | 50 | 50 nM → 390 pM in duplicate, serial 2x dilutions, randomly injected | | 300 | 300 | Kinetics | |  |
|  | 10 | 2 | 84 | 700 | |  |  |  |  |  | 1:1 binding | |  |

**Supplementary Table 4 | SPR methodology and calculated TEAD interaction parameters of optides.** The reported association constants for TB1G2 and TB1G2-W40P are approaching the limits of resolution for the instrument. ND: Equilibrium measurements were used to characterize these interactions because kinetic analyses (association / dissociation constants) could not be accurately fitted. Sequences show color-coded mutations from TB1G1. Alterations in red reduce TEAD binding; those in blue improve TEAD binding. hTFR(ED): Human transferrin receptor ectodomain.

| **Construct and Read Direction** | **Full Sanger sequencing results** |
| --- | --- |
| TB1G1  Forward | NNNNNNNNNTNNNNNNNCTTNTCAAGAGCCTGGGGCTGCCCGAGGAATCATATTGTGTTCCCAGTCCCCATCGACCAGTGTATTGATGGAGGCGGGAGTGAAAACCTGTACTTTCAG**GGATCCCCTGATGAATATATTGAACGCGCCAAAGAATGCTGCAAAAAAGGCGATATTCAGTGCTGCCTGCGCTATTTCGAAGAATCCGGGGACCCCAACGTGATGCTGATTTGCCTGTTCTGCCCCTAATGCGGCCGC**TCATCACCATTAATCATCACCATTAATCGGACCGCCTCTCCCTCCCCCCCCCCTAACGTTACTGGCCGAAGCCGCTTGGAATAAGGCCGGTGTGCGTTTGTCTATATGTTATTTTCCACCATATTGCCGTCTTTTGGCAATGTGAGGGCCCGGAAACCTGGCCCTGTCTTCTTGACGAGCATTCCTAGGGGTCTTTCCCCTCTCGCCAAAGGAATGCAAGGTCTGTTGAATGTCGTGAAGGAAGCAGTTCCTCTGGAAGCTTCTTGAAGACAAACAACGTCTGTAGCGACCCTTTGCAGGCAGCGGAACCCCCCACCTGGCGACAGGTGCCTCTGCGGCCAAAAGCCACGTGTATAAGATACACCTGCAAAGGCGGCACAACCCCAGTGCCACGTTGTGAGTTGGATAGTTGTGGAAAGAGTCAAATGGCTCTCCTCAAGCGTATTCAACAAGGGGCTGAAGGATGCCCAGAAGGTACCCCATTGTATGGGATCTGATCTGGGGCCTCGGTGCACATGCTTTACATGTGTTTAGTCGAGGTTAAAAAAACGTCTAGGCCCCCCGAACCACGGGGACGTGGTTTTCCTTTGAAAAACACGATGATAAGCTTGCCACAACCCTTATCGAGGCCACCATGGTGAGCAAGGGCGAGGAGCTGTTCACCGGGGTGGTGCCCATCCTGGTCGAGCTGGACGGCGACGTAAACGGCCACAAGTTCAGCGTGTCCGGCGAGGGCGAGGGCGATGCCNNCTACGGCAAGCTGACCNTGAAGTTCATCTGCNCACNGCAAGCTGCCCGTGCCCTNNNNCCTCNNGACNNCCTGACNACGGNGNGCANTGCTCANCGCTNCCCGACNCATGAAGCANCACGACTTNNNAAGTCNNN |
| TB1G1-L37A  Forward | NNNNNNNNNTNCNNNNNNNNTTTCAAGAGCCTGGGGCTGCCCGAGAATCATATTGTGTTCCCAGTCCCCATCGACCAGTGTATTGATGGAGGCGGGAGTGAAAACCTGTACTTTCAG**GGATCCCCAGATGAATATATTGAGCGCGCAAAAGAATGCTGCAAAAAAGGTGATATTCAGTGCTGCCTGCGCTATTTCGAGGAATCTGGGGACCCTAACGTGATGCTGATTTGCGCCTTCTGCCCCTAATGCGGCCGC**TCATCACCATTAATCATCACCATTAATCGGACCGCCTCTCCCTCCCCCCCCCCTAACGTTACTGGCCGAAGCCGCTTGGAATAAGGCCGGTGTGCGTTTGTCTATATGTTATTTTCCACCATATTGCCGTCTTTTGGCAATGTGAGGGCCCGGAAACCTGGCCCTGTCTTCTTGACGAGCATTCCTAGGGGTCTTTCCCCTCTCGCCAAAGGAATGCAAGGTCTGTTGAATGTCGTGAAGGAAGCAGTTCCTCTGGAAGCTTCTTGAAGACAAACAACGTCTGTAGCGACCCTTTGCAGGCAGCGGAACCCCCCACCTGGCGACAGGTGCCTCTGCGGCCAAAAGCCACGTGTATAAGATACACCTGCAAAGGCGGCACAACCCCAGTGCCACGTTGTGAGTTGGATAGTTGTGGAAAGAGTCAAATGGCTCTCCTCAAGCGTATTCAACAAGGGGCTGAAGGATGCCCAGAAGGTACCCCATTGTATGGGATCTGATCTGGGGCCTCGGTGCACATGCTTTACATGTGTTTAGTCGAGGTTAAAAAAACGTCTAGGCCCCCCGAACCACGGGGACGTGGTTTTCCTTTGAAAAACACGATGATAAGCTTGCCACAACCCTTATCGAGGCCACCATGGTGAGCNAAGGGCGAGGAGCTGTTCACCGGGGTGGTGCCCATCCTGGNCGAGCTGGACGGCGACGTAAACGGCCACAAGTTCAGCGTGTCCGGCGAGGGCNANGGNCGATGCCANCTACGGCAGCTGACCCTGAAGTNATCTGCACNNNGNNNCTGCCCGTGCCNNNNNNNCCNCNNGACNNCNGACTANNGNNNGCNNGNNTNNNCNNTNCCGACNNNTNANCANNANGANTNNNNAGTCNCNNNNCNNNNNTACNTCNNGN |
| TB1G1-F38A  Forward | NNNNNNNNNNNNNNNNNNNTTTCAAGAGCCTGGGGCTGCCCGAGAATCATATTGTGTTCCCAGTCCCCATCGACCAGTGTATTGATGGAGGCGGGAGTGAAAACCTGTACTTTCAG**GGATCCCCTGATGAATATATTGAACGCGCCAAAGAATGCTGCAAAAAAGGCGATATTCAGTGCTGCCTGCGCTATTTCGAAGAAAGTGGTGACCCCAACGTGATGCTGATTTGCCTGGCCTGCCCCTAATGCGGCCGC**TCATCACCATTAATCATCACCATTAATCGGACCGCCTCTCCCTCCCCCCCCCCTAACGTTACTGGCCGAAGCCGCTTGGAATAAGGCCGGTGTGCGTTTGTCTATATGTTATTTTCCACCATATTGCCGTCTTTTGGCAATGTGAGGGCCCGGAAACCTGGCCCTGTCTTCTTGACGAGCATTCCTAGGGGTCTTTCCCCTCTCGCCAAAGGAATGCAAGGTCTGTTGAATGTCGTGAAGGAAGCAGTTCCTCTGGAAGCTTCTTGAAGACAAACAACGTCTGTAGCGACCCTTTGCAGGCAGCGGAACCCCCCACCTGGCGACAGGTGCCTCTGCGGCCAAAAGCCACGTGTATAAGATACACCTGCAAAGGCGGCACAACCCCAGTGCCACGTTGTGAGTTGGATAGTTGTGGAAAGAGTCAAATGGCTCTCCTCAAGCGTATTCAACAAGGGGCTGAAGGATGCCCAGAAGGTACCCCATTGTATGGGATCTGATCTGGGGCCTCGGTGCACATGCTTTACATGTGTTTAGTCGAGGTTAAAAAAACGTCTAGGCCCCCCGAACCACGGGGACGTGGTTTTCCTTTGAAAAACACGATGATAAGCTTGCCACAACCCTTATCGAGGCCACCATGGTGAGCAAGGGCGAGGAGCTGTTCACCGGGGTGGTGCCCATCCTGGTCGAGCTGGACGGCGACGTAAACGGCCACAAGTTCAGCGTGTCCGGCGAGGGCGAGGGCGATGCCACCTACGGCAAGCTGACCCTGAAGTTCATCTGCACCACCGGCAAGCTGCCCGTGCCCTNNNCNNCCTCGTGACNNNN |
| TB1G2  Reverse | CNNNNNNNNNNNNGNNNNNNNNNNNNNNNGTTNGGGGGGGGGGAGGGAGAGGCGGTCCGATTNATGGTGATGATTAATGGTGATGA**GCGGCCGCATTACCAGCAGAACAGGCAAATCAGCATCACGTTGGGATCTTTGCTTTCATCGAAAATGCGCAGGCAGCACTGAATATCCTGTTTTTTGCAGCATTCTTTGGCGCGTTCAATATATTCATCGGGGGATCC**CTGAAAGTACAGGTTTTCACTCCCGCCTCCATCAATACACTGGTCGATGGGGACTGGGAACACAATATGATTCTCGGGCAGCCCCAGGCTCTTTGAAAAGCGGATGAAGTTTTCTTTCAGCTCGCTAGTCAGTTCCTTTGTTCTGCCATACAGGGTGATCTTGAAGTACTCCCGATTCTGGCTGACCTTTTTAAAGAACACCATAGCGTGCTGATTATAGTTGGTAGAGACCACTCGCACCAGATAACTAGTCAGCCCTGGGTAAGACTTAATGTTGCCCAGAGTAAACTCTCCTGGCTGGCTCCCAGGCACGAATGTCCTGATCCAATAATCGCACTTTTTCTTTCTGAACAGGACAGAAGTCACATTGTAGGACTTGTCTTCTTTCAGCTCGTAGATTGTGGCGTACATCTTCTGAGGATCTTTGTCCTCTCTCAGGATAGCGTTCCCGGCCAGGCCGACCACGTACCATTTGCCCTGAAACTGATTGTCCTGGAAGTTCTGCTGCAGAGGCACCTTACTCAGAGGGGGTGCGGGGATCAGATCACTGGTAGAGTCCTGAGACCCGCCATGGTGATGATGATGATGTTCGTCTTTGTAGTCCCCAGTTGAGCCGGGCACCCACAGCAGCAGCACCCACAGCAGCAGAGTATCGGTTTCCATGGTCTCGAGCGGGCGACTCAGTCTGTCNGAGGACTGGCGCGCCGAGTGAGGGGTTGTGAGCTCTTTTATAGAGCTCGGGAAGCAGANCGCGCGAACAGAAGCGAGAAGCNNNTGATTNNNNATTCAANANGNGCAGGTCATTTCNNNCCTTGGGGGAGCCTGNAAACNTNCNNGATGGGNCTTAANAAACTGCTGAGGNNGGGCCANNANTNNGGG |
| TB1G2-W40P  Reverse | NNNNNNNNNNNNNNGNNNNNNNNNNNNNNANNGTTAGGGGGGGGGGAGGGAGAGGCGGTCCGATTAATGGTGATGATTAANGGTGANGA**GCGGCCGCATTAGGGGCAGAACAGGCAAATCAGCATCACGTTGGGATCTTTGCTTTCATCGAAAATGCGCAGGCAGCACTGAATATCCTGTTTTTTGCAGCATTCTTTGGCGCGTTCAATATATTCATCGGGGGATCC**CTGAAAGTACAGGTTTTCACTCCCGCCTCCATCAATACACTGGTCGATGGGGACTGGGAACACAATATGATTCTCGGGCAGCCCCAGGCTCTTTGAAAAGCGGATGAAGTTTTCTTTCAGCTCGCTAGTCAGTTCCTTTGTTCTGCCATACAGGGTGATCTTGAAGTACTCCCGATTCTGGCTGACCTTTTTAAAGAACACCATAGCGTGCTGATTATAGTTGGTAGAGACCACTCGCACCAGATAACTAGTCAGCCCTGGGTAAGACTTAATGTTGCCCAGAGTAAACTCTCCTGGCTGGCTCCCAGGCACGAATGTCCTGATCCAATAATCGCACTTTTTCTTTCTGAACAGGACAGAAGTCACATTGTAGGACTTGTCTTCTTTCAGCTCGTANATTGTGGCGTACATCTTCTGAGGATCTTTGTCCTCTCTCAGGATAGCGTTCCCGGCCAGGCCGACCACGTACCATTTTGCCCNTGANANNGATTGNCCTGNAGTTCTGCTGCAGAGGCACCTTACTCAGANGGGGTGCGGGGATCAGATCACTGGTAGAGNCCTGAGACCCGCCATGGTGA |

**Supplementary Table 5 | Raw Sanger sequencing data for Daedalus-expressed TEAD binding optides.** Cloned optide constructs between upstream BamHI (GGATCC) and downstream NotI (GCGGCCGC) sites are in bold, underlined font. Raw .ab1 trace files are available as **Supplementary Dataset 1**.

**Supplementary Methods: High Diversity Native CDP Library Data Processing**

**Note:** The data to which this methodology was applied is available as **Supplementary Data 3.**

**Sample collection description**

Definitions:

“Run”: One of the two experimental repeats of the entire screen.

“Condition”: One of the two treatments (untreated or trypsin) per run.

“Sample”: Per condition, cells were sorted into one of four tubes based on APC signal, from the anti-6xHis antibody stain. Each of those sorted tubes is a “sample”; there were four samples from each of the two conditions, in addition to an “input” sample (cells were transduced and un-sorted) and an “apc-high” sample (cells were sorted by APC+, which were later subjected to PBS or trypsin treatment), for ten samples total per run. However, most of the statistical analysis was limited to the 8 samples from the two treatment conditions.

“Replicate”: All samples were grown post-sort (or post-transduction for the input) to amplify the cell population. After growth, tubes of cells (1.5 million each) were collected for multiplex sequencing. Each collected tube is a “replicate”; there were three replicates per sample, so there were a total of 30 replicates per run.

The ten samples (with three technical replicates each) were collected as follows:

1) input: pellets from the transduced cells with no sorting applied.

2) apc_high: pellets from cells that were stained with 8 nM AlexaFluor 647-conjugated anti-6xHis, and the cells collected had APC staining above that of untransduced cells; i.e. detectible peptide on the cell surface.

untreated: apc_high cells were grown, and then on the day of the sort, were split in half. This half was treated sequentially with PBS, DTT (10 mM), and 8 nM AlexaFluor 647-conjugated anti-6xHis.

3) untreated_highest: during the untreated sort, the >90 percentile of APC stained cells (i.e. the top 10%) were collected in this sample.

4) untreated_high: during the untreated sort, the ~68-88 percentile of APC stained cells (i.e. the next 20%, with ~2% bin separation) were collected in this sample.

5) untreated_low: during the untreated sort, the ~46-76 percentile of APC stained cells (i.e. the next 30%, with ~2% bin separation) were collected in this sample.

6) untreated_lowest: during the untreated sort, the bottom ~34 percentile of APC stained cells (i.e. the remainder, with ~2% bin separation) were collected in this sample.

trypsin: apc_high cells were grown, and then on the day of the sort, were split in half. This half was treated sequentially with trypsin (5 µg/mL in PBS), DTT (10 mM), and 8 nM AlexaFluor 647-conjugated anti-6xHis.

7) trypsin_highest: during the trypsin treated sort, the >90 percentile of APC stained cells(i.e. the top 10%) were collected in this sample.

8) trypsin_high: during the trypsin treated sort, the ~68-88 percentile of APC stained cells (i.e. the next 20%, with ~2% bin separation) were collected in this sample.

9) trypsin_low: during the trypsin treated sort, the ~46-76 percentile of APC stained cells (i.e. the next 30%, with ~2% bin separation) were collected in this sample.

10) trypsin_lowest: during the trypsin treated sort, the bottom ~34 percentile of APC stained cells (i.e. the remainder, with ~2% bin separation) were collected in this sample.

During the untreated / trypsin treated sort, two statistics were collected on the flow cytometer (seen on the fluorescence_stats tab):

1) The actual sorted proportion per bin (used to adjust cell counts). The guidelines for the desired distributions are above, but the numbers used for data processing were the actual stats from the sort.

2) The median APC channel fluorescence for the whole population (used to normalize between the two runs) and for each bin (to quantitate gene-by-gene surface protein content).

Three technical replicates (techrep1-3) were performed in the form of three tubes of 1.5 million cells that were pelleted, and PCR-amplified with unique barcodes, for each of the 10 samples per run.

The 1.5 million cells per replicate required growing of the cells after the sort until total population was ~10-20 million cells per sample, to allow for three replicates plus backup pellets .

The experiment was repeated twice (run1 and run2). Each run used the same library at the plasmid pool level, but new virus was made and new cells were transduced for each run.

For each run, two lanes of Illumina HiSeq (rapid run mode) were run, with 6 base barcodes for multiplexing.

Lane 1 samples were: input, apc_high, untreated_lowest, untreated_low, and untreated_high.

Lane 2 samples were: untreated_highest, and all four trypsin samples.

**Statistical processing**

raw_read_counts: All Illumina-sequenced samples were split by barcode, and mapped (bowtie2) to the original oligonucleotide library, with default scoring (except for discarding all frameshifted reads). Note: any genes that fail to produce any reads in any of the four conditions are eliminated.

reads_per_million: raw_read_counts within each replicate were summed, with the sum used to normalize each gene's read counts (per million mapped reads). It is at this point that the two thresholding values can be calculated (see below).

cell_proportion_adjusted: reads_per_million were multiplied by that sample's “cell_fraction” (e.g. ~10 for "highest" samples), found on the "fluorescence_stats" tab. This provides a proportional estimate of the cells carrying the given gene within each sample. For each peptide, a sum of its 12 replicates per condition are calculated for normalization (see next).

cell_per_condition_norm: To normalize for gene abundance within the total untreated or trypsin treated libraries (i.e. get "fluorescence per gene per 100 cells" not "fluorescence per gene in the condition as a whole"), the cell_proportion_adjusted values were divided by the sum of that condition's twelve replicates' cell_proportion_adjusted values. These values were then multiplied by 100, to give a value per gene that approximates the gene-by-gene percentage distribution amongst the condition’s twelve replicates. So, within a condition, the sum of the 12 samples’ values after this normalization should be 100.

fluor_per_bin: cell_per_condition_norm values were multiplied by that sample's median fluorescence (as a percentage of the full untreated sort's un-binned median fluorescence), found on the "fluorescence_stats" tab as “apc_norm_to_apc+”. This approximates the total surface protein content of cells expressing a given gene within each sample/replicate. Normalizing to the full sort's APC+ fluorescence value was done to be able to compare the two runs, which were sorted on different cytometers with different PMT voltages.

protein_content: the untreated and trypsin treated protein content per condition is the sum of the twelve replicates' fluor_per_bin values for each condition / run, producing four content values per gene. Final values were the average of the two runs for a given treatment (untreated or trypsin). These values produced the two key statistics:

1. Untreated protein content (X axis of Fig. 2b):
2. Trypsin resistance (Y axis of Fig. 2b; trypsin protein content as a % of untreated protein content).

Note that, because all of these values are estimates based on sequencing data that is inherently variable, it is possible for a peptide's trypsin protein content to calculate as higher than the untreated content. This can result in a peptide's caluclated trypsin resistance being > 100%.

**Read abundance thresholds**

Values were calculated for all genes, but two thresholds were applied to reduce variance as a result of small read counts for a given gene. The values chosen were arbitrary, but yielded data that correlated well between the two runs.

1) A gene's reads per million must exceed 50 in at least one of the three apc_high replicates, in both of the two runs.

2) A gene's reads per million must exceed 100 in at least one of the twelve sorted replicates (four samples, three replicates each) in the untreated condition, in both of the two runs.

The columns "apchi_rpm_threshold" and "untreated_rpm_threshold" represent these values:

apchi_rpm_threshold =

min(max(run1_apchi_techrep[1,2,3]),max(run2_apchi_rechrep[1,2,3]))

untreated_rpm_threshold =

min(max(run1_untreated_[all 12 samples]),max(run2_untreated_[all 12 samples]),))

Genes that pass both of these thresholds (i.e. "apchi_rpm_threshold" > 50, "untreated_rpm_threshold" > 100) were included in final comparative statistical analysis.

**Assignment of peptides to various categories**

High protein content / trypsin resistant (HPTR) vs low protein content / trypsin sensitive (LPTS)

An arbitrary border was used to separate peptides into high protein content and/or trypsin resistant, or low protein content and/or trypsin sensitive. PC = Untreated Protein Content. TR = trypsin resistance.

The calculation for this is:

X = TR - (150 - PC/400)

If X > 0, peptide is marked as high protein content / trypsin resistant, or "HPTR".

If X < 0, peptide is marked as low protein content / trypsin sensitive, or "LPTS".

Knottin or defensin (here, simplified as "knottin")

All knottin sequences were downloaded from the knottin database, at knottin.cbs.cnrs.fr/

All peptides were mapped to these sequences. Anything that maps is marked as a knottin.

Additionally, all peptides whose gene description contains the word "defensin" or "Defensin" is marked as a knottin.

Then, all of these knottin sequences were converted to their cysteine topology (all non-C converted to X, and all X outside of flanking C's eliminated).

Then, all peptides in the library were converted to their cysteine topology, and any peptides whose topology matched one on the knottin topology list were also marked as a knottin.

Proportion of full protein length

UniProt IDs were searched within the UniProt database to generate the consensus full-length protein sequence.

Peptide matches were confirmed, and then each peptide's length as a fraction of the length of the full sequence was calculated.

Note: not all peptides have a consensus UniProt full length sequence. These may have been part of UniProt database builds that were later removed. These peptides were kept in the library to increase diversity, but were omitted for any analysis that included protein proportions.

Glycosite

All peptide sequences were searched for "NXS" or "NXT". Any with at least one motif found were flagged as glycosite-containing.
