# Supplementary material for: Mammalian display screening of diverse cystine-dense peptides for difficult to drug targets
Source: Nat Commun. 2017 Dec 21;8:2244. doi: 10.1038/s41467-017-02098-8 (PMC5740061; doi:10.1038/s41467-017-02098-8)
Supplement: Supplementary file 3 — Description of Additional Supplementary Files [file 41467_2017_2098_MOESM3_ESM.pdf]

Description of Additional Supplementary Files

File Name: Supplementary Data 1
Description: **Raw Sanger sequencing trace files for Daedalusexpressed TEAD binding optides**. Found in text form in Supplementary Table 4, this data was used to confirm the presence of the properly cloned, sequence-validated TEAD binding optides (TB1G1, TB1G1-L37A, TB1G1-F38A, TB1G2, and TB1G2- W40P) for production as soluble peptides.

File Name: Supplementary Data 2
Description: **TB1G1 SSM sort 2 enrichment values.** Presented here in tabular format is the data found in the heat map in Figure 5b. The values represent the enrichment scores for every possible non-Cys substitution in TB1G1, tested for TEAD binding in SDGF surface display screening (20 nM, monovalent staining). Rows are amino acid substitutions, grouped by chemical category. Columns are the TB1G1 protein sequence, duplicated below the heat map. Enrichment score represents a variant's fold-change in population abundance after two rounds of TEAD sorting versus its input abundance, normalized to TB1G1, and log2-transformed.

File Name: Supplementary Data 3
Description: **High throughput protein content data.** This is the raw and processed high throughput sequencing data used for analysis of surface protein content of the high diversity CDP library, presented in summarized form in Figure 2, Figure 3, and Supplementary Table 3. The Supplementary Methods provide a detailed guide for the generation, processing, and interpretation of this data. The sequences, in the form of DNA or protein for each CDP, were withheld as proprietary information.
